# DCO-MAC: A Hybrid MAC Protocol for Data Collection in Underwater Acoustic Sensor Networks

**DOI:** 10.3390/s18072300

**Published:** 2018-07-16

**Authors:** Min Deng, Huifang Chen, Lei Xie

**Affiliations:** 1 College of Information Science and Electronic Engineering, Zhejiang University, Hangzhou 310027, China; dengminzju@zju.edu.cn (M.D.); xiel@zju.edu.cn (L.X.); 2 Zhejiang Provincial Key Laboratory of Information Processing, Communication and Networking, Hangzhou 310027, China

**Keywords:** underwater acoustic sensor networks (UASNs), medium access control (MAC), data-collection-oriented MAC (DCO-MAC) protocol, hybrid MAC protocol

## Abstract

In underwater acoustic sensor networks (UASNs), medium access control (MAC) is an important issue because of its potentially significant effect on the network performance. However, designing a suitable MAC protocol for the UASN is challenging because of the specific characteristics of the underwater acoustic channel and network, such as limited available bandwidth, long propagation delay, high bit-error-rate, and sparse network topology. In addition, as the traffic load is non-uniformly distributed in a UASN for data collection, it is essential to consider the application feature for the MAC protocol. In this paper, we propose a MAC protocol in a data-collection-oriented UASN, abbreviated as the DCO-MAC protocol. In the proposed protocol, the network is partitioned into two kinds of sub-networks according to the traffic load. A contention-based MAC protocol is used in the sub-network with a light traffic load, while a reservation-based MAC protocol is used in the sub-network with a heavy traffic load. Meanwhile, the DCO-MAC protocol supports the access of mobile nodes. The theoretical analysis and simulation results demonstrate that, in a UASN for data collection, the proposed MAC protocol outperforms the other existing MAC protocols, in terms of the network throughput, end-to-end packet delay, energy overhead, and fairness.

## 1. Introduction

In the last decade, underwater acoustic sensor networks (UASNs) have attracted enormous research attention in the academia and industry [1,2,3], as UASNs can be used in many applications, such as ocean currents monitoring, and oceanographic data collection, submarine detection, and so on. Unlike terrestrial wireless sensor networks, radio and light are not suitable for underwater communication because of the severe attenuation in this environment. Hence, sound wave is the preferred choice for the underwater communication scenario.

Designing an appropriate medium access control (MAC) protocol is important for the UASN. In this paper, we focus on the MAC protocol in the UASN for data collection mission. However, the special characteristics of the underwater acoustic channel and network, such as long propagation delay, high bit-error-rate (BER), limited bandwidth, and sparse network topology, pose a great challenge to underwater MAC protocol design. Moreover, as an application-oriented network, the MAC protocol designing in the UASN should take the requirements of the applications into consideration.

In this paper, we consider a data-collection-oriented UASN (DCO-UASN), consisting of a sink node (SN) and many sensor nodes. In this network, the data packets generated at the sensor nodes are transmitted to the SN hop-by-hop. Thus, the traffic load in the DCO-UASN is non-uniformly distributed. That is, the closer to the SN, the more data packets are transmitted by the sensor node.

In recent years, many MAC protocols have been proposed in the UASNs. These MAC protocols can be classified into three types, contention-based, schedule-based, and hybrid MAC protocols. In contention-based MAC protocols [4,5,6,7,8,9,10], sensor nodes need to compete for the channel to send data packets. The contention-based MAC protocol for the UASN achieves a small end-to-end delay in the case of a low network traffic load. However, the successful probability of the channel competition decreases rapidly as the network traffic load increases, which results in the decrease of the network throughput. In order to achieve a high network throughput, the schedule-based MAC protocols have been proposed for UASNs [11,12,13]. In the schedule-based MAC protocols, the transmissions of senders are scheduled to avoid the packet collisions. Hence, the schedule-based MAC protocols in the UASN achieve a high network throughput with a high network traffic load. However, the end-to-end delay of the schedule-based MAC protocols is large, especially when the network traffic load is low. In addition, the non-uniformly distribution of the network traffic load in the DCO-UASN is not considered in both the contention-based and schedule-based MAC protocols.

Combining the advantages of the contention-based and schedule-based MAC protocols, the hybrid MAC protocols [14,15] are adaptive to the network traffic load by providing multiple modes, where different protocols are adopted in different modes. One finds, in the work of Shahabudeen and Chitre [14], that the hybrid MAC protocol performs well in the single-hop DCO-UASN. However, the sender–receiver collision occurs when the protocol is extended directly to multi-hop DCO-UASN, because some nodes may carry out several MAC protocols in the same time. In the work of Lotfinezhad et al. [16], a hybrid MAC protocol is proposed for a cluster-based DCO-UASN, where the intra-cluster communication and inter-cluster communication are carried out periodically, to avoid the sender–receiver collision. However, in this protocol, a contention-based MAC protocol is adopted for the inter-cluster communication with a high network load, which results in a poor network throughput performance. Therefore, we have studied the hybrid MAC protocol for the DCO-UASN with a non-uniformly distributed traffic load in this work.

On the other hand, with the development of autonomous underwater vehicle (AUV) technology, AUV is widely used in oceanographic data collection. However, the access of the mobile node will influence the transmission of the fixed nodes. Thus, we need consider the transmission of both the mobile and fixed nodes in the MAC protocol designing. There are some MAC protocols proposed for the UASN with mobile nodes [17,18]. In the work of Yoon and Qiao [17], according to the real-time location information of the mobile node, the sink node schedules the transmission time slot of the sensor node dynamically, in order to avoid transmission collisions. A code division multiple access (CDMA)-based MAC protocol is proposed by Khan and Cho [18], in order to avoid the transmission collision, where different orthogonal spread codes are allocated to different mobile nodes. However, the CDMA technique introduces the near–far problem.

In this paper, we propose a MAC protocol for a two-tier DCO-UASN. In the proposed MAC protocol, the network is partitioned into two kinds of sub-networks according to the traffic load, and the suitable MAC protocols are designed for the different kinds of sub-networks. For the sub-network with a light traffic load, a ConTention-based MAC (CT-MAC) protocol is carried out. In the proposed CT-MAC protocol, when one of the sensor nodes having data packet(s) to be transmitted reserves the channel, the receiving node will give a response to the received channel reservation control packet, as well as inviting other sensor nodes having data packet(s) to be transmitted in the same sub-network, to join in the transmission process. For the sub-network with a heavy traffic load, a ReSerVation-based MAC (RSV-MAC) protocol is performed. In the RSV-MAC protocol, the receiver initiates the transmission process. To avoid the transmission collision and achieve a high throughput, the receiver pre-schedules the transmission of both the control packets and data packets. Furthermore, in order to support the access of mobile nodes, the extra transmission time is reserved in both the CT-MAC and RSV-MAC protocols.

The remainder of the paper is structured as follows. In Section 2, we review the related work about MAC protocols in UASNs. The system model is described in Section 3. In Section 4, we present a hybrid MAC protocol in the UASN for data collection, in detail. Section 5 gives the performance analysis of the proposed MAC protocol in the renewal theory. Numerical results and discussions are given in Section 6. Finally, we conclude the paper in Section 7.

## 2. Related Work

The MAC protocols for the UASNs can be divided into three types, namely the contention-based MAC protocols, the scheduled-based MAC protocols, and the hybrid MAC protocols. In the contention-based MAC protocols, the sensor nodes with data packets to be transmitted first require exchanging the control packet with the receiver to reserve the channel. Multiple access collision avoidance (MACA) [5] is a typical contention-based MAC protocol. In MACA, the sender transmits a request-to-send (RTS) packet to inform the receiver to get ready to receive data packets. After receiving the RTS packet correctly, the receiver responds with a clear-to-send (CTS) packet to inform the sender of transmitting data packets. Other nodes receiving the RTS or CTS packets should be silent for some time, to avoid the packet transmission collisions. Because of the long propagation delay in the UASNs, it is time consuming to reserve a channel using the RTS/CTS packets. Moreover, only one sender is allowed to transmit the data packet(s) after a long-time channel reservation, and the throughput of the MACA protocol is poor in UASNs.

In order to improve the throughput, several modified MAC protocols are proposed, based on the MACA protocol, such as bidirectional-concurrent MAC protocol (BIC-MAC) [8], adaptive propagation delay tolerant collision avoidance protocol (APCAP) [9], and multiple handshaking MAC protocol (MHM) [10]. In BIC-MAC, to achieve a higher throughput, once the sender reserves the channel successfully, the sender and receiver transmit data packets to each other in the same time slot first, and then turn into the receiving mode. Considering the long waiting time before receiving the CTS packets from the receiver, the APCAP allows the sender to perform other operations during the waiting time, so as to improve the throughput. In the MHM, the receiver can receive multiple RTS packets from several senders in the same time slot, and respond with a CTS packet to all of these senders in the next time slot. Meanwhile, the receiver schedules the transmission of the data packets to avoid the transmission collision caused by multiple senders, and to improve the network throughput. All of these modified MAC protocols achieve a higher throughput in some extent, compared with the MACA. However, with the increasing traffic load, the collision probability of the RTS packets increases, and the throughput of the contention-based MAC protocols therefore decreases rapidly.

Some schedule-based MAC protocols are proposed for the UASNs with a high traffic load. In the path-oriented CDMA-based MAC (POCA-CDMA-MAC) protocol [11], the SN builds the data transmission paths for the sensor nodes in the network and assigns a different spreading sequence to each path. The nodes in the same path transmit in a round-robin manner, each node, except the first sender, has to receive the data packets from its previous node before sending the packets. The packets are encoded with a spreading sequence assigned to that path, so that the packets from multiple paths can be received by SN simultaneously without collision, which results in a higher throughput. However, the CDMA brings a high system complexity, and its near–far problem is hard to overcome. Handshake-based ordered scheduling MAC protocol (HOSM) [12] and receiver-initiated packet train MAC protocol (RIPT) [13] are both receiver-initiated MAC protocols for UASNs, the receiver schedules the transmission of the RTS packets in order to realize the handshaking with multiple senders concurrently. Then, the receiver schedules the data packet transmission of the senders, using the information of propagation delay, so that the data packets can be received in a packet train manner. The schedule-based MAC protocols avoid the collision of the control packets and achieve a higher throughput in the UASNs with a high traffic load. However, in a receiver-initiated MAC protocol, the receiver has no idea whether the sender having data packets needs to send or not. In a UASN with a low traffic load, the probability that no senders respond to the transmission initiated by the receiver is high, which results in the performance degradation.

The performance of the contention-based and schedule-based MAC protocols are poor when the traffic load distribution in the network is non-uniform. In order to achieve a good performance in such a situation, some hybrid MAC protocols are proposed for the UASNs. In the adaptive multi-mode MAC protocol (MAC-AMM) [14], multiple protocols are incorporated. When the traffic load is low, a random-access MAC protocol is performed. As the traffic load increases, a contention-based or a reservation-based MAC protocol is chosen according to the network architecture. Underwater practical MAC protocol (UPMAC) [15] chooses suitable MAC protocols based on the traffic load. In UPMAC, ALOHA is performed when the network works in a low traffic load mode, in which the sensor nodes transmit the data packets to the receiver without channel reservation, and the number of data packets intended to transmit to the receiver is included in the data packets. The receiver extracts this information and performs a reservation-based MAC protocol if the number of data packets exceeds the threshold. The hybrid MAC protocols achieve a good performance in the single-hop underwater sensor networks. However, when these hybrid MAC protocols extended to a multi-hop underwater sensor network, a node may carry out several protocols simultaneously, and transmission collision may be caused.

Several MAC protocols intended for multi-hop underwater sensor networks are also proposed. The cluster-based MAC protocol (CH-MAC) [16] and cluster-based data collection schemes with direct sink access (CDS-DSA) [19] are both designed for a clustered UASN. In both of the MAC protocols, the transmission is partitioned into two phases, namely the intra-cluster communication phase and the inter-cluster communication phase. The transmission of the two phases is executed periodically, in order to avoid the transmission collision. In the intra-cluster communication, time division multiple access (TDMA) is carried out in these two MAC protocols. In the inter-cluster communication, ALOHA and CSMA/CA are used in CDS-DSA and CH-MAC, respectively. CDS-DSA and CH-MAC are energy-efficient, and achieve a low end-to-end packet delay. However, if the traffic load of the inter-cluster communication is heavier than that of the intra-cluster communication, ALOHA and CSMA/CA will lead to high collision probability in the inter-cluster communication.

In an underwater mobile network, the SN or sensor nodes can be a mobile node. CC-DCP [20] is a TDMA-based MAC protocol used in a UASN, in which the SN is a mobile node. The SN travels along a pre-scheduling path to collect the data packets of the static sensor nodes. In order to improve the performance, the SN communicates with multiple sensor nodes in one transmission, and schedules the transmission time slot of the sensor nodes to avoid collision. MC-MAC [18] is another MAC protocol that is used in a UASN, in which the sensor nodes are mobile. The sensor node, having data packets to be transmitted, moves to the SN to initiate transmission, and the CDMA technique is used to avoid the transmission collision.

## 3. System Model

**Figure 1:**
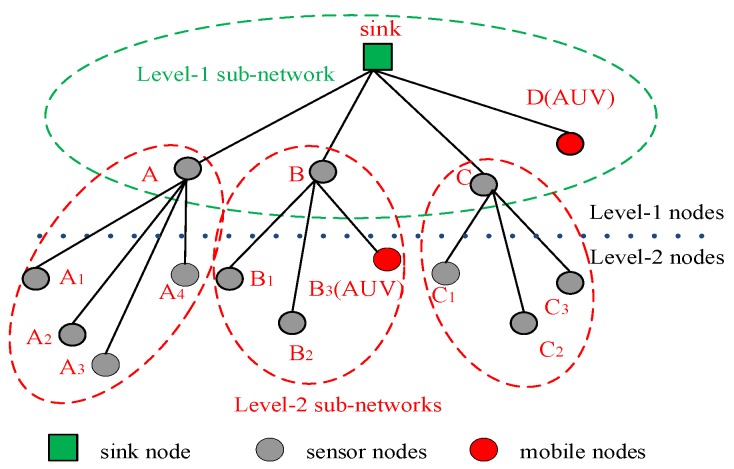
The network topology of the two-tier underwater acoustic sensor network (UASN) for data collection.

In this paper, we consider a two-tier UASN for data collection, all of the sensor nodes are in the two-hop transmission range of SN, and the network topology is shown in Figure 1.

Sensor nodes are classified into three types, namely, the sink, level-1 nodes, and level-2 nodes. The level-1 nodes are in the one-hop transmission range of the sink, such as A, B, C and D in Figure 1. Level-2 nodes are in the two-hop transmission range of the sink, such as A_1_, A_2_, and B_3_, in Figure 1. In addition, the mobile nodes are included in the system model, such as D and B_3_, in Figure 1. The mobile nodes join in the network only when they have data packet(s) to be transmitted. Because of the low movement speed of the mobile nodes, the change of their locations is small in the period of one data transmission. Hence, we assume that the position change of the mobile nodes will not influence the performance of the MAC protocols. Hence, the position change of the mobile nodes is neglected in our proposed MAC protocols.

Because of the sparse distribution of the sensor nodes in the UASN, we assume that the level-1 nodes are not in the transmission range of the other level-1 nodes. The mobile nodes move along the designed path to collect data and transmit data packets to a level-1 node or the sink. All of the other sensor nodes float slightly with the ocean current around the fixed position.

The data packet transmission path of each node is determined after the network is deployed, and each sensor node has one corresponding receiver. After being deployed, the sink broadcasts a path formation massage. The sensor nodes receiving the path formation massage from the sink are regarded as level-1 nodes, and their common receiver is the sink. Then, the level-1 nodes broadcast path formation messages. The sensor nodes receiving path formation messages from the level-1 nodes are regarded as level-2 nodes, and determine their corresponding receivers. If a level-2 node receives more than one path formation message, it selects the level-1 node, whose path formation message arrives earlier, to be its receiver. In this way, the data transmission path from each sensor node to the sink is determined.

The DCO-UASN is partitioned into two kinds of sub-networks [21]. The level-1 sub-network consists of the sink and several level-1 nodes, such as A, B, C, and D in Figure 1. The level-2 sub-network consists of one level-1 node and several level-2 nodes. For example, a level-2 sub-network includes nodes A, A_1_, A_2_, A_3_, and A_4_ in Figure 1.

In the DCO-UASN, the data packets generated by the sensor nodes are transmitted from the sensor nodes to the SN. From Figure 1, the level-1 nodes not only transmit the generated data packets, but also forward the data packets received from the corresponding level-2 nodes to the sink. Obviously, the traffic load in the level-1 sub-network is heavier than that in the level-2 sub-network. However, as most of the existing MAC protocols are designed for underwater sensor networks with a uniform distribution of the traffic load, they cannot be utilized to the DCO-UASN directly. Therefore, it is essential to design a suitable MAC protocol in the DCO-UASN. In this paper, we propose a hybrid MAC protocol for data collection in the UASN, where different sub-networks perform different MAC protocols according to their traffic load.

## 4. Proposed DCO-MAC Protocol

In the network initialization phase, the position of the sensor nodes is obtained by performing a localization algorithm. The sensor nodes broadcast HELLO packets to exchange position information. After receiving HELLO packets from the neighboring nodes, the sensor nodes calculate the propagation delay with the neighboring nodes according to the position information; the propagation delay is calculated as the ratio of the distance and sound speed. In the system model described in Section 3, a level-1 node belongs to a level-1 sub-network and one of the level-2 sub-networks. In order to avoid the collision, the data packet transmission in two kinds of sub-networks is executed alternately.

As the traffic load is heavy in the level-1 sub-network, the probability that multiple level-1 nodes simultaneously send control packets to reserve the channel is relatively high, which results in a high collision possibility at the sink. Hence, a RSV-MAC protocol is implemented in the level-1 sub-network. In the RSV-MAC protocol, the sink initiates the transmission of the level-1 sub-network, and schedules the data transmission of the level-1 nodes to avoid the collision. Because of the light traffic load in the level-2 sub-network, the collision probability of the control packets is small. Therefore, a CT-MAC protocol is carried out in the level-2 sub-network, where the level-2 node sends a control packet to reserve the channel before sending the data packets. In addition, in order to support the access of the mobile nodes, extra transmission time is reserved in both the RSV-MAC and CT-MAC protocols.

### 4.1. RSV-MAC Protocol

The RSV-MAC is a receiver-initiated MAC protocol.

In the transmission phase of the level-1 sub-network, the sink broadcasts a RTR (ready to receive) packet to initiate the transmission, and waits to receive the ATS (available to send) packets transmitted by the level-1 nodes. In order to avoid the collision of the ATS packets, the sink calculates the time period between the RTR packet sending and the ATS packet receiving, *T*_wtr_ATS,*i*_, based on the propagation delay between the sink and the level-1 node, *i*, and specifies the pre-scheduling time in the RTR packet. When receiving the RTR packet, the level-1 node, *i*, send an ATS packet after waiting for a period, *T*_w_ATS,*i*_, to guarantee its ATS packet reaching the sink at the pre-scheduled time. After receiving all of the ATS packets from the level-1 nodes, the sink calculates the time period the between the ORDER packet sending and the DATA packets receiving from level-1 node, *i*, *T*_wtr_DATA,*i*_, and broadcasts the waiting time by an ORDER packet. After receiving the ORDER packet, the level-1 node, *i*, sends the DATA packet(s) after waiting a period, *T*_w_DATA,*i*_, in order to guarantee its DATA packet(s) being received by the sink at the pre-scheduled time. Finally, after receiving all of the data packets, the sink broadcasts an ACK packet to notify the completion of the transmission phase in the level-1 sub-network.

**Figure 2:**
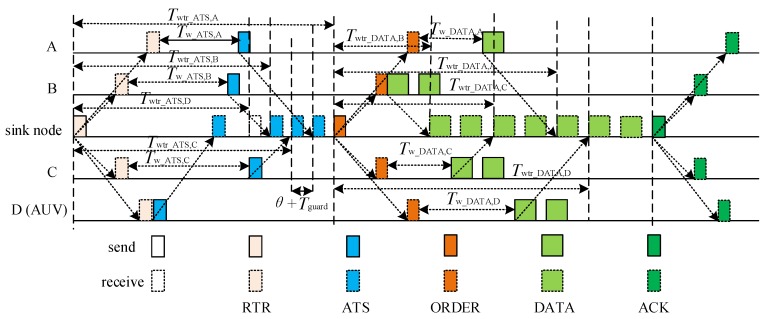
The process of data transmission in ReSerVation-based medium access control (RSV-MAC) protocol.

Figure 2 shows the process of data transmission in RSV-MAC protocol.

The sink pre-schedules the transmission of the ATS packets from the level-1 nodes. To minimize the receiving period of the ATS packets, the sink pre-schedules to receive them in an increasing order of the propagation delay between the sink and the level-1 nodes. However, as the position of the mobile node is not fixed, the propagation delay between the sink and a mobile node is unknown. In order to support the access of the mobile node, the propagation delay between the sink and a mobile node is assumed to be the maximum propagation delay in the UASN, *τ*_max_. The ATS packet of a mobile node is scheduled to be first received by the sink. As shown in Figure 2, the receiving order of the ATS packets is D→B→C→A. To mitigate the impact of the node movement with the ocean current on the transmission, a guard time, *T*_guard_, is added between the two consecutive ATS packets. Let *τ_i,j_* denote the propagation delay between node *i* and node *j*.

According to Figure 2, the time period between the RTR packet sending and the ATS packet receiving from node *i*, *T*_wtr_ATS,*i*_, is given as follows:

*T*_wtr_ATS,*i*_ = *T*_wtr_ATS,D_ + (*O*_ATS,*i*_ − 1)(*θ* + *T*_guard_), *i* ∈ {A, B, C}, (1)

where *θ* is the transmission delay of control packet, and *O*_ATS,*i*_ denotes the transmission order of the ATS packet from node *i*.

The time period between the RTR packet sending and the ATS packet receiving from mobile node D is *T*_wtr_ATS,D_ = 2*τ*_max_ + *θ*. Meanwhile, the receiving period of the ATS packets at the sink can be calculated as *T*_r_ATS,sink_ = *T*_wtr_ATS,A_ + *θ* + *T*_guard_.

The sink broadcasts the RTR packet containing the time period between the RTR packet sending and the ATS packet receiving.

When receiving the RTR packet, the level-1 nodes without the data packet to be transmitted keep silent. Otherwise, the level-1 nodes with data packets to be transmitted extract the time period between the RTR packet sending and the ATS packet receiving from the RTR packet, and transmit the ATS packet to the sink after waiting a period. The ATS packet contains the number of data packets to be transmitted, *φ_i_*, at the level-1 node, *i*. According to Figure 2, the waiting period at node *i*, *T*_w_ATS,*i*_, can be set as follows:

*T*_w_ATS,*i*_ = *T*_wtr_ATS,*i*_ − 2*τ*_sink,*i*_ − *θ*, *i* ∈ {A, B, C}. (2)

The mobile node is scheduled to send the ATS packet once it receives the RTR packet successfully. Therefore, the waiting time of node D is assumed to be *T*_w_ATS,D_ = 0. After receiving the ATS packet from the mobile node, the propagation delay between the sink and the mobile node can be calculated by the sink, using the RTR packet sending time and ATS packet receiving time. Hence, the mobile node can be treated as a normal one in the follow-up data transmission phase.

According to the increasing order of propagation delay between the sink and level-1 nodes, the transmission order of the DATA packets is B→C→A→D, as illustrated in Figure 2. After receiving the ATS packets from the level-1 nodes, the sink calculates the time period between the ORDER packet sending and the DATA packets receiving from node *i*, *T*_wtr_DATA,*i*_, as follows:

(3) $$T_{\mathrm{wtr}\_\mathrm{DATA},i}=T_{\mathrm{wtr}\_\mathrm{DATA},B}+\sum_{\begin{array}{l} k\in \{A,B,C,D\}\\ O_{\mathrm{DATA},k}<O_{\mathrm{DATA},i} \end{array}}^{}\varphi_{k}(\delta +T_{\mathrm{guard}}),\;i\in \{A,C,D\}$$

where *δ* is the transmission delay of the DATA packet, and *O*_DATA,*i*_ denotes the transmission order of the DATA packet at node *i*.

The time period between the ORDER packet sending and the DATA packets receiving, from the first node in the transmission order, node B, is *T*_wtr_DATA,B_ = 2*τ*_sink,B_ + *θ*. Meanwhile, the receiving period of the DATA packets at the sink can be calculated as *T*_r_DATA,sink_ = *T*_wtr_DATA,D_ + *φ*_D_(*δ + T*_guard_).

The sink broadcasts the ORDER packet containing {*T*_wtr_DATA,*i*_|*i* ∈ {A,B,C,D}} and *T*_r_DATA,sink_.

After receiving the ORDER packet, the level-1 node, *i*, with data packet(s) to be transmitted, sends the DATA packet(s) to the sink node after waiting a period, *T*_w_DATA,*i*_. The waiting period at node *i*, *T*_w_DATA,*i*_, can be set as follows:

*T*_w___DATA,*i*_ = *T*_wtr_DATA,*i*_ − 2*τ*_sink,*i*_ − *θ*, *i* ∈ {A, B, C, D}. (4)

After receiving the DATA packets successfully, the sink broadcasts an ACK packet to finish the transmission of the level-1 sub-network.

**Figure 3:**
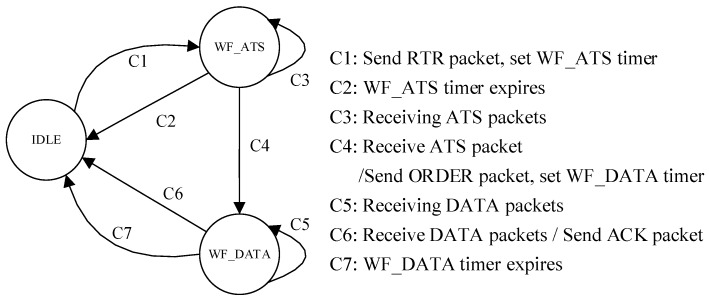
The state transition diagram of the sink. RTR—ready to receive; ATS—available to send; WF_ATS—wait for ATS.

In the RSV-MAC protocol, the sink has three states, namely, IDLE, WF_ATS (wait for ATS), and WF_DATA (wait for DATA). The state transition diagram of the sink is shown in Figure 3.

The sink starts from the IDLE state at the beginning of level-1 sub-network transmission phase. The sink broadcasts the RTR packet, sets a WF_ATS timer, and enters the WF_ATS state. The duration of the WF_ATS timer is *T*_WF_ATS_timer_ = *T*_r_ATS,sink_.

When the WF_ATS timer expires, and no ATS packets are received, the sink goes back to the IDLE state. When an ATS packet is received, and the receiving phase of the ATS packets are not finished, the sink keeps the current state. When all of the pre-scheduled ATS packets are received or the WF_ATS timer expires, the sink broadcasts the ORDER packet, sets a WF_DATA timer, and enters the WF_DATA state. The duration of the WF_DATA timer is *T*_WF_DATA_timer_ = *T*_r_DATA,sink_.

When the WF_DATA timer expires, and no DATA packets are received, the sink goes back to the IDLE state. When a DATA packet is received, and the receiving phase of the DATA packets does not finish, the sink keeps the current state. When all of the pre-scheduled DATA packets are received or the WF_DATA timer expires, the sink broadcasts the ACK packet, and goes back to the IDLE state.

**Figure 4:**
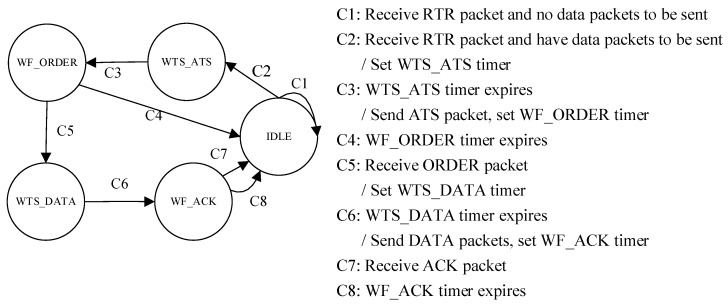
The state transition diagram of level-1 node in the RSV-MAC protocol.

The level-1 node has five states in RSV-MAC protocol, namely IDLE, WTS_ATS (wait to send ATS), WF_ORDER (wait for ORDER), WTS_DATA (wait to send DATA), and WF_ACK (wait for ACK). The state transition diagram of the level-1 node in the RSV-MAC protocol is shown in Figure 4.

The initial state of the level-1 node is the IDLE state. When receiving the RTR packet, the level-1 node without data packets to be transmitted keeps silent in the current state. Otherwise, the level-1 node with the data packet(s) to be transmitted sets the WTS_ATS timer and enters the WTS_ATS state. The duration of the WTS_ATS timer is *T*_WTS_ATS_timer_ = *T*_wtr_ATS,*i*_ − 2*τ*_sink,*i*_ *− θ*.

When the WTS_ATS timer expires, the level-1 node sends its ATS packet to the sink at the pre-scheduled time, sets the WF_ORDER timer, and enters the WF_ORDER state. The duration of the WF_ORDER timer is *T*_WF_ORDER_timer_ = *T*_r_ATS,sink_ − *T*_w_ATS,*i*_.

When the WF_ORDER timer expires, and the ORDER packet is not received, the level-1 node goes back to the IDLE state. When receiving the ORDER packet, the level-1 node sets the WTS_DATA timer and enters the WTS_DATA state. The duration of the WTS_DATA timer is *T*_WTS_DATA___timer_ = *T*_wtr_DATA,*i*_ − 2*τ*_sink*,i*_ − *θ*.

When the WTS_DATA timer expires, the level-1 node transmits the DATA packet(s) to the sink at the pre-scheduled time, sets the WF_ACK timer, and enters the WF_ACK state. The duration of the WF_ACK timer is *T*_WF_ACK_timer_ = *T*_r_DATA,sink_ − *T*_w_DATA,*i*_.

When the WF_ACK timer expires, and the ACK packet is not received, the level-1 node goes back to the IDLE state. Otherwise, the ACK packet is received before the WF_ACK timer expires, the level-1 node gets the corresponding information from the ACK packet, and it goes back to the IDLE state.

### 4.2. CT-MAC Protocol

The CT-MAC is a contention-based MAC protocol using the handshaking mechanism for channel reservation.

In the beginning of the transmission phase of the level-2 sub-network, the level-2 nodes with data packet(s) to be transmitted send the RTS packet to the reserve channel. After receiving an RTS packet successfully, the level-1 node broadcasts an INVITE packet to respond to the received RTS packet and invite other level-2 nodes in the same sub-network to join in the transmission process. To avoid the collision, the level-1 node calculates the time period between the INVITE packet sending and the NOTICE packet receiving, according to the propagation delay between the level-1 node and level-2 nodes, *T*_wtr_NOTICE,*k*_. In addition, the time period between the INVITE packet sending and the NOTICE packet receiving are included into the INVITE packet. Receiving the INVITE packet, the level-1 node *k* with data packet(s) to be transmitted sends the NOTICE packet after waiting a period, *T*_w_NOTICE,*k*_, to guarantee that the NOTICE packets are reaching the level-1 node at the pre-scheduled time. After receiving all of the NOTICE packets, the level-1 node calculates the time period between the ORDER packet sending and the DATA packets receiving, from the level-2 nodes that have transmitted a NOTICE packet, *T*_wtr_DATA,*k*_, and broadcasts an ORDER packet including these calculated time periods. Receiving the ORDER packet, the level-2 nodes with the data packet(s) to be transmitted send the DATA packet(s) after waiting a period, *T*_w_DATA,*k*_, so as to guarantee that the DATA packet(s) are being received by the level-1 node at the pre-scheduled time. Finally, after receiving all of the data packets, the level-1 node broadcasts an ACK packet.

**Figure 5:**
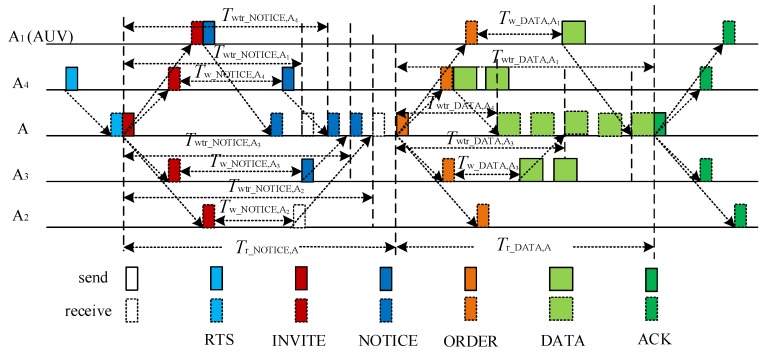
The process of data transmission in the contention-based MAC (CT-MAC) protocol.

Figure 5 illustrates the process of data transmission in CT-MAC protocol.

In a level-2 sub-network, receiving an RTS packet from one of the level-2 nodes with data packet(s) to be transmitted, the level-1 node pre-schedules the transmission of the NOTICE packets from the level-2 nodes. The time period between the INVITE packet sending and the NOTICE packet receiving can be calculated in the same way as the time period between the RTR packet sending and the ATS packet receiving in the RSV-MAC protocol. As shown in Figure 5, the receiving order of the NOTICE packets is A_1_→A_4_→A_3_→A_2_. The time period between the INVITE packet sending and the NOTICE packet receiving from node *k*, *T*_wtr_NOTICE,*k*_, can be calculated as follows:

(5) $$T_{\mathrm{wtr}\_\mathrm{NOTICE},k}=T_{{\mathrm{wtr}\_\mathrm{NOTICE},A}_{1}}+(O_{\mathrm{NOTICE},k}-1)(\theta +T_{\mathrm{guard}}),k\in \{A_{2}{,A}_{3}{,A}_{4}\}$$

where *O*_NOTICE,*k*_ denotes the transmission order of the NOTICE packet from node *k*.

The time period between the INVITE packet sending and the NOTICE packet receiving from the mobile node A_1_ is $T_{{\mathrm{wtr}\_\mathrm{NOTICE},A}_{1}}=2\tau_{\max}+\theta$. The total receiving period of the NOTICE packets at node A is $T_{r\_\mathrm{NOTICE},A}=T_{{\mathrm{wtr}\_\mathrm{NOTICE},A}_{2}}+\theta +T_{\mathrm{guard}}$.

The level-1 node in a level-2 sub-network (node A) broadcasts the INVITE packet containing the calculated time period between the INVITE packet sending and the NOTICE packet receiving.

When receiving the INVITE packet, the level-2 node without a data packet to be transmitted keeps silent. Otherwise, the level-2 node with data packets to be transmitted extracts the NOTICE receiving time from the INVITE packet, and transmit the NOTICE packet to the level-1 node after waiting a period, *T*_w_NOTICE,*k*_. The NOTICE packet contains the number of data packets to be transmitted, *φ_k_*, at the level-2 node, *k*. From Figure 5, the waiting period at node *k*, *T*_w_NOTICE,*k*_, can be set as follows:

*T*_w_NOTICE,*k*_ = *T*_wtr_NOTICE,*k*_ − 2*τ*_A,*k*_ − *θ*, *k* ∈ {A_2_, A_3_, A_4_}. (6)

The mobile node is scheduled to send the NOTICE packet once it receives the INVITE packet successfully. So, the waiting time of the mobile node A_1_ is $T_{w\_\mathrm{NOTICE},A_{1}}=0$. After receiving the NOTICE packet from a mobile node, the propagation delay between the level-1 node (A) and the mobile node (A_1_) can be calculated by node A, using the NOTICE packet sending time and the NOTICE packet receiving time. Then, the mobile node can be treated as a normal one in the follow-up data transmission phase.

According to the increasing order of propagation delay between node A and the level-2 nodes, the transmission order of the DATA packets is A_4_→A_3_→A_1_, as illustrated in Figure 5. After receiving the NOTICE packets from the level-2 nodes with the data packet(s) to be transmitted, node A calculates the time period between the ORDER packet sending and the DATA packets receiving from node *k*, *T*_wtr_DATA,*k*_, as follows:

(7) $$T_{\mathrm{wtr}\_\mathrm{DATA},k}=T_{{\mathrm{wtr}\_\mathrm{DATA},A}_{4}}+\sum_{\begin{array}{l} m\in \{A_{1}{,\;A}_{3}{,\;A}_{4}\}\\ O_{\mathrm{DATA},m}<O_{\mathrm{DATA},k} \end{array}}^{}\varphi_{m}(\delta +T_{\mathrm{guard}}),k\in \{A_{1}{,A}_{3}{,A}_{4}\}\;$$

where the time period between the ORDER packet sending and the DATA packets receiving from the first node in the transmission order, node A_4_, is $T_{{\mathrm{wtr}\_\mathrm{DATA},A}_{4}}=2\tau_{{A,A}_{4}}+\theta$. Meanwhile, the total receiving period of the DATA packets at node A is $T_{r\_\mathrm{DATA},A}=T_{{\mathrm{wtr}\_\mathrm{DATA},A}_{1}}+\varphi_{A_{1}}\left(\delta +T_{\mathrm{guard}}\right)$.

Node A broadcasts an ORDER packet containing {*T*_wtr_DATA,*k*_|*i* ∈ {A_1_, A_3_, A_4_ }} and *T*_r_DATA,A_.

After receiving the ORDER packet, the level-2 node, *k*, with the data packet(s) to be transmitted, send the DATA packet(s) to node A after waiting a period, *T*_w_DATA,*k*_. The waiting period at node *k*, *T*_w_DATA,*k*_, can be set as follows:

*T*_w_DATA,*k*_ = *T*_wtr_DATA,*k*_ − 2*τ*_A*,k*_ − *θ*, *k* ∈ {A_1_, A_3_, A_4_}. (8)

After receiving the DATA packets successfully, the level-1 node A broadcasts an ACK packet to finish the transmission of the level-2 sub-network.

If a level-2 node is in the transmission range of multiple level-2 sub-networks, we name this kind of node an interference node. If an interference node receives an *x*INVITE or *x*ORDER packet, it means that the neighboring level-2 sub-network is under the transmission process. So, the interference node should keep silent until the completion of the neighboring sub-network data transmission, to avoid the interference. The silent time is (2*N*_neighbor_ + 2) *τ*_max_, where *N*_neighbor_ is the number of level-2 nodes in the corresponding neighbor level-2 sub-network, and the value of *N*_neighbor_ can be extracted from the *x*INVITE or *x*ORDER packet. If an *x*RTS or *x*NOTICE is received, the interference node should keep silent for 3*τ*_max_, to avoid interference, according to Figure 5. As the level-1 nodes charge the data transmission of a level-2 sub-network, the level-1 nodes do not back off, even if they receive a control packet not intended to them, in order to avoid the interference on the data transmission of multiple level-2 nodes.

**Figure 6:**
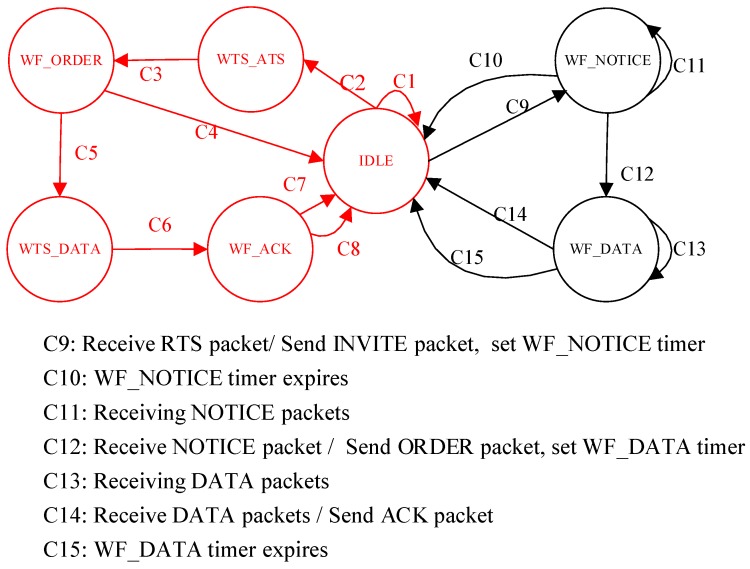
The whole state transition diagram of a level-1 node in the data-collection-oriented (DCO)-MAC protocol.

In the CT-MAC protocol, the level-1 node has three states, namely IDLE, WF_NOTICE (waif for NOTICE), and WF_DATA (wait for DATA). As a level-1 node belongs to a level-1 sub-network and one of level-2 sub-networks simultaneously, the whole state transition diagram of a level-1 node in the DCO-MAC protocol is shown in Figure 6, where the part in red has been explain in Figure 4.

The level-1 node starts from the IDLE state at the beginning of the level-2 sub-network transmission phase. When the level-1 node receives an RTS packet from one of its level-2 nodes successfully, it broadcasts an INVITE packet, sets the WF_NOTICE timer, and enters the WF_NOTICE state. The duration of the WF_NOTICE timer is *T*_WF_NOTICE_timer_ = *T*_r_NOTICE,A_.

When the WF_NOTICE timer expires and no NOTICE packets are received, te level-1 node goes back to the IDLE state. When a NOTICE packet is received and the receiving period of the NOTICE packets is not finished, the level-1 node keeps the current state. When all of the pre-scheduled NOTICE packets are received or the WF_NOTICE timer expires, the level-1 node broadcasts an ORDER packet, sets the WF_DATA timer, and enters the WF_DATA state. The duration of the WF_DATA timer is *T*_WF_DATA_timer_ = *T*_r_DATA,A_.

When the WF_DATA timer expires and no DATA packets are received, the level-1 node goes back to the IDLE state. When a DATA packet is received and the receiving phase of the DATA packets is not finished, the level-1 node keeps the current state. When all of the pre-scheduled DATA packets are received or the WF_DATA timer expires, the level-1 node broadcasts the ACK packet, and goes back to the IDLE state.

**Figure 7:**
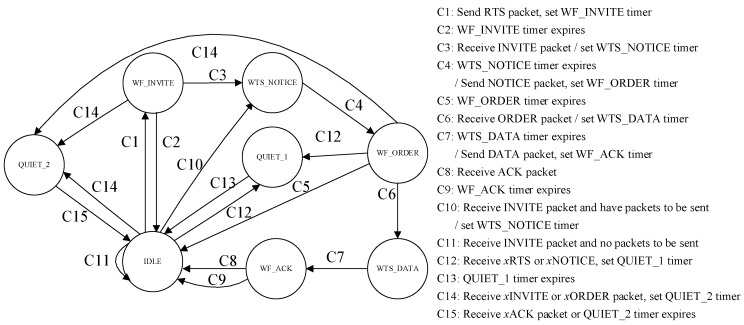
The state transition diagram of a level-2 node in CT-MAC protocol.

The level-2 node has eight states in the CT-MAC protocol, namely IDLE, WTS_NOTICE (wait to send NOTICE), WF_INVITE (wait for INVITE), WF_ORDER (wait for ORDER), WTS_DATA (wait to send DATA), WF_ACK (wait for ACK), QUIET_1, and QUIET_2. The state transition diagram of a level-2 node in the CT-MAC protocol is shown in Figure 7.

The initial state of the level-2 node is the IDLE state. In the beginning, the level-2 node without a data packet to be transmitted keeps silent in the current state. The level-2 node with data packet(s) to be transmitted sends an RTS packet to reserve the channel, sets the WF_INVITE timer, and enters the WF_INVITE state. The duration of the WF_INVITE timer is *T*_WF_INVITE_timer_ = 2*τ*_A,*k*_ + 2*θ*.

When the WF_INVITE timer expires and the INVITE packet is not received, the level-2 node goes back to the IDLE state. When receiving the INVITE packet, the level-2 node with data packet(s) to be transmitted sets the WTS_NOTICE timer and enters the WTS_NOTICE state. The duration of the WTS_NOTICE timer is *T*_WTS_NOTICE_timer_ = *T*_wtr_NOTICE,*k*_ − 2*τ*_A,*k*_ *− θ.*

When the WTS_NOTICE timer expires, the level-2 node sends its NOTICE packet to the level-1 node at the pre-scheduled time, sets the WF_ORDER timer, and enters the WF_ORDER state. The duration of the WF_ORDER timer is *T*_WF_ORDER_timer_ = *T*_r_NOTICE,A_ − *T*_w_NOTICE,*k*_.

When the WF_ORDER timer expires and the ORDER packet is not received, the level-2 node goes back to the IDLE state. When receiving the ORDER packet, the level-2 node sets the WTS_DATA timer and enters the WTS_DATA state. The duration of the WTS_DATA timer is *T*_WTS_DATA_timer,*k*_ = *T*_wtr_DATA,*k*_ − 2*τ*_A*,k*_ − *θ*.

When the WTS_DATA timer expires, the level-2 node transmits the DATA packet(s) to the level-1 node at the pre-scheduled time, sets the WF_ACK timer, and enters the WF_ACK state. The duration of the WF_ACK timer is *T*_WF_ACK_timer_ = *T*_r_DATA,A_ − *T*_w_DATA,*k*_.

When the WF_ACK timer expires and the ACK packet is not received, the level-2 node goes back to the IDLE state. Otherwise, if the ACK packet is received before the WF_ACK timer expires, the level-2 node gets the corresponding information from the ACK packet and goes back to the IDLE state.

When level-2 node with data packet(s) to be transmitted receives an *x*RTS or *x*NOTICE packet, it sets the QUIET_1 timer, and enters the QUIET_1 state. The duration of QUIET_1 timer is *T*_QUIET_1_timer_ = 3*τ*_max_. When the QUIET_1 timer expires, the level-2 node goes back to the IDLE state.

When the level-2 with data packet(s) to be transmitted receives *x*INVITE or *x*ORDER packet, it sets the QUIET_2 timer, and enters the QUIET_2 state. The duration of the QUIET_2 timer is *T*_QUIET_2_timer_ = (2*N*_neighbor_ + 2)*τ*_max_. When the *x*ACK packet is received or the QUIET_2 timer expires, the level-2 node goes back to the IDLE state.

In the DCO-MAC protocol, the transmission of the level-1 sub-network and level-2 sub-network is performed alternatively. In a data collection period, the CT-MAC protocol is first carried out in the level-2 sub-networks, the level-1 nodes broadcast a short message to the level-2 nodes to inform them about the start of the level-2 transmission phase, and the sink is in the IDLE state. In each level-2 sub-network, the *N*_trans_ times data collection are performed during the data transmission phase of the level-2 sub-network. After the *N*_trans_ times data collection is finished, the corresponding level-1 node sends a short message to the sink and waits for the response message. If the response message is not received, the level-1 node sends the short message again, until it receives the response message. After receiving the short messages from all the level-1 nodes, the sink broadcasts an RTR packet to the level-1 nodes, which means that the procedure of the RSV-MAC protocol starts. During a data collection period, the RSV-MAC protocol is carried out at the same time. In the end of the data transmission of the level-1 sub-network, the level-1 nodes announce a short message to inform the new data collection period.

In this paper, we assume that there is only one mobile node in each sub-network. However, the proposed MAC protocol can be extended to the network with one more mobile node. If several mobile nodes exist in the network, their transmission order should be set before their deployment. Then, the sink or level-1 node reserves 2*τ*_max_ time for each mobile node to receive the ATS or NOTICE packet. After receiving the RTR or INVITE packets, the mobile node, *I*, waits 2*τ*_max_(*O*_mobile,*i*_ − 1) and sends an ATS or NOTICE packet, where *O*_mobile,*i*_ is the transmission order of the mobile node, *i*. The sink or level-1 node calculates the propagation delay of the mobile nodes after receiving the ATS or NOTICE packet.

## 5. Approximate Performance Analysis

According to the renewal theory [22], the average channel utilization can be given as the ratio of the average data transmission time and total transmission time. That is, as follows:

(9) $$S=\frac{\bar{U}}{\bar{B}+\bar{I}}\;$$

where $\bar{B}$ is the expected time duration of a busy period when the channel is being utilized. $\bar{I}$, the expected time duration of an idle period, denotes the time interval between two consecutive busy periods. $\bar{U}$ denotes the expected time duration when the channel is used to transmit the data packets.

### 5.1. CT-MAC Protocol

**Figure 8:**
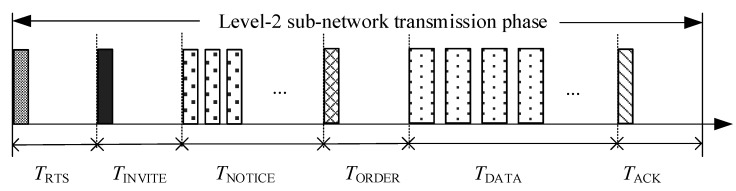
The transmission period of CT-MAC protocol.

In the CT-MAC protocol, a successful data collection period, *T*_SUC_, is composed of a successful transmission of the RTS packet, INVITE packet, several NOTICE packets, and ORDER packet, followed by a data transmission period and a successful transmission of the ACK packet. The transmission period of the CT-MAC protocol is illustrated in Figure 8.

If the bit-error-rate of the underwater acoustic channel is high, the retransmission of the DATA packets is required when a packet error occurs. Therefore, in the level-2 sub-network, *j*, the transmission time of hte DATA packet can be calculated as follows:

(10) $$T_{\mathrm{DATA}}=\tau_{\max}+\sum_{i=0}^{\infty }iK_{\mathrm{level}-2}(\delta +T_{\mathrm{guard}})p_{e}^{i-1}(1-p_{e})=\tau_{\max}+\frac{K_{\mathrm{level}-2}(\delta +T_{\mathrm{guard}})}{1-p_{e}}\;$$

Hence, *T*_SUC_ is given as follows:

(11) $$T_{\mathrm{SUC}}=T_{\mathrm{RTS}}+T_{\mathrm{INVITE}}+T_{\mathrm{NOTICE}}+T_{\mathrm{ORDER}}+T_{\mathrm{DATA}}+T_{\mathrm{ACK}}=\;6\tau_{\max}+(N_{j}+4)\theta +N_{j}T_{\mathrm{guard}}+\frac{K_{\mathrm{level}-2}(\delta +T_{\mathrm{guard}})}{1-p_{e}},$$

where *K*_level-2_, the number of DATA packets transmitted in a level-2 sub-network during *T*_SUC_, can be calculated as *K*_level-2_ = *N_j_λ*(*τ*_max_ + *θ*); if the Poisson arrival is assumed, *N_j_* is the number of level-2 nodes in the considered level-2 sub-network; *λ* is the packet arrival rate at each level-2 node; and *p*_e_ is the packet error rate.

For a successful channel reservation with the RTS packet, only one level-2 node is allowed to send an RTS packet during *T*_RTS_, and the RTS packet transmission of other level-2 nodes and interference nodes should be avoided. Therefore, the probability of a successful channel reservation is as follows:

(12) $$P_{\mathrm{SUC}}=\mathrm{Pr}(\mathrm{only}\;\mathrm{one}\;\mathrm{RTS}\;\mathrm{arrival}\;\mathrm{in}\;T_{\mathrm{RTS}}|\mathrm{RTS}\;\mathrm{arrivals}\;\mathrm{in}\;T_{\mathrm{RTS}}\;\mathrm{period})\;=\;\;\frac{N_{j}\lambda (\tau_{\max}+\theta )e^{-\lambda (N_{j}+N_{\mathrm{int},j})(\tau_{\max}+\theta )}}{1-e^{-N_{j}\lambda (\tau_{\max}+\theta )}},$$

where *N*_int,*j*_ is the number of interference nodes in level-2 sub-network, *j*.

A failed transmission period, *T*_FAIL_, is the length of an RTS transmission period. That is, as follows:

*T*_FAIL_ = *T*_RTS_ = *τ*_max_ + *θ*. (13)

A busy period can be a period of a successful or a failed data transmission. Hence, $\bar{B}$ is given as follows:

(14) $$\bar{B}=T_{\mathrm{SUC}}P_{\mathrm{SUC}}+T_{\mathrm{FAIL}}(1-P_{\mathrm{SUC}})\;$$

The expected time duration of the channel being used to transmit the DATA packets is given as follows:

(15) $$\bar{U}=K_{\mathrm{level}-2}\delta P_{\mathrm{SUC}}.$$

According to the definition of the Poisson distribution, the probability that no RTS packets are generated in *T*_RTS_ is *P*_0_ = $e^{-N_{j}\lambda (\tau_{\max}+\theta )}$. The expected time duration of the idle period equals the expected time duration without the generated RTS packets. Hence, $\bar{I}$ can be calculated as follows:

(16) $$\bar{I}=\sum_{i=0}^{\infty }\left(ie^{-N_{j}\lambda i(\tau_{\max}+\theta )}(\tau_{\max}+\theta )\right)=\frac{\tau_{\max}+\theta }{1-e^{-N_{j}\lambda i(\tau_{\max}+\theta )}}\;$$

Substituting Equations (14)–(16) into Equation (9), the normalized throughput of the level-2 sub-network, *j*, can be calculated as follows:

(17a) $$S_{\mathrm{net}2,j}=\frac{\delta P_{\mathrm{SUC}}N_{j}\lambda (\tau_{\max}+\theta )}{\left[6\tau_{\max}+(N_{j}+4)\theta +\frac{N_{j}\lambda (\tau_{\max}+\theta )(\delta +T_{\mathrm{guard}})}{1-p_{e}}\right]P_{\mathrm{SUC}}+(\tau_{\max}+\theta )(1-P_{\mathrm{SUC}})+\frac{\tau_{\max}+\theta }{1-e^{-N_{j}\lambda (\tau_{\max}+\theta )}}}$$

(17b) $$P_{\mathrm{SUC}}=\frac{N_{j}\lambda (\tau_{\max}+\theta )e^{-\lambda (N_{j}+N_{\mathrm{int},j})(\tau_{\max}+\theta )}}{1-e^{-N_{j}\lambda (\tau_{\max}+\theta )}}$$

### 5.2. RSV-MAC Protocol

**Figure 9:**
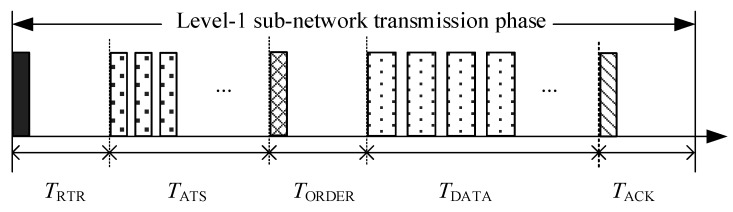
The transmission period of RSV-MAC protocol.

In the RSV-MAC protocol, a successful data transmission period, *T*_SUC_, is composed of a successful transmission of an RTR packet, several ATS packets, and an ORDER packet, followed by a data transmission period and the successful transmission of an ACK packet. The transmission period of CT-MAC protocol is illustrated in Figure 9.

In the level-1 sub-network transmission phase, *T*_SUC_ can be calculated as follows:

(18) $$T_{\mathrm{SUC}}=T_{\mathrm{RTS}}+T_{\mathrm{ATS}}+T_{\mathrm{ORDER}}+T_{\mathrm{DATA}}+T_{\mathrm{ACK}}=\;5\tau_{\max}+(N_{\mathrm{level}-1}+3)\theta +N_{\mathrm{level}-1}T_{\mathrm{guard}}+\frac{K_{\mathrm{level}-1}(T_{\mathrm{guard}}+\delta )}{1-p_{e}},$$

where *N*_level-1_ is the number of nodes in the level-1 sub-network, and *K*_level-1_ is the number of DATA packets transmitted in the level-1 sub-network in *T*_SUC_.

In the level-1 sub-network, the level-1 nodes not only transmit the data packets generated by themselves to the sink, but also forward the gathered data packets from the corresponding level-2 nodes. Hence, the probability that no data packets are sent in the level-1 sub-network transmission phase is very small. To simplify the calculation, we assume that the level-1 nodes want to transmit data packets at the beginning of the level-1 sub-network transmission phase. Hence, the data transmission in the level-1 sub-network is assumed to be successful. So, the busy period can be given as follows:

(19) $$\bar{B}=T_{\mathrm{SUC}}\;$$

There are two types of data packets sent by the level-1 nodes, one is generated by the level-1 nodes during the last data transmission period, the other is gathered from the level-2 nodes during the last level-2 sub-network data transmission phase. Hence, *K*_level-1_ can be given as follows:

(20) $$K_{\mathrm{level}-1}=N_{\mathrm{level}-1}\lambda (\bar{B}+\bar{I})+\varphi_{\mathrm{level}-2}\;$$

where *φ*_level-2_, the expected number of data packets gathered from the level-2 sub-network by level-1 nodes, can be given as follows: $\varphi_{\mathrm{level}-2}=N_{\mathrm{trans}}\sum_{j=1}^{N_{\mathrm{net}2}}P_{\mathrm{SUC},j}N_{j}\lambda (\tau_{\max}+\theta )$.

Combining Equations (18), (19), and (20), *K*_level-1_ can be rewritten as follows:

(21) $$K_{\mathrm{level}-1}=\frac{N_{\mathrm{level}-1}\lambda \left[5\tau_{\max}+(N_{\mathrm{level}-1}+3)\theta +N_{\mathrm{level}-1}T_{\mathrm{guard}}+\bar{I}\right]+\varphi_{\mathrm{level}-2}}{1-\frac{N_{\mathrm{level}-1}\lambda (T_{\mathrm{guard}}+\delta )}{1-p_{e}}}\;$$

As *K*_level-1_ is always greater than 0, we have $\frac{N_{\mathrm{level}-1}\lambda (T_{\mathrm{guard}}+\delta )}{1-p_{e}}<1$ in Equation (21), where $\frac{T_{\mathrm{guard}}+\delta }{1-p_{e}}$ is the expected receiving time of one DATA packet, and $\frac{N_{\mathrm{level}-1}\lambda (T_{\mathrm{guard}}+\delta )}{1-p_{e}}$ denotes the number of data packets that are generated by the level-1 nodes during $\frac{T_{\mathrm{guard}}+\delta }{1-p_{e}}$. Hence, $\frac{N_{\mathrm{level}-1}\lambda (T_{\mathrm{guard}}+\delta )}{1-p_{e}}<1$ indicates that the data packet arrival rate of the level-1 nodes should be less than the data packet transmission rate. Otherwise, the network will be congested, and cannot operate normally.

The expected time duration of the channel being used to transmit the DATA packets can be given as follows:

(22) $$\bar{U}=K_{\mathrm{level}-1}\delta .$$

The expected time duration of idle period equals the transmission time of the level-2 sub-networks. That is, as follows:

(23) $$\bar{I}=\underset{j}{\max}N_{\mathrm{trans}}\left(\left[6\tau_{\max}+(N_{j}+4)\theta +\frac{N_{j}\lambda (\tau_{\max}+\theta )(T_{\mathrm{guard}}+\delta )}{1-p_{e}}\right]P_{\mathrm{SUC},j}+(\tau_{\max}+\theta )(1-P_{\mathrm{SUC},j})\right)\;$$

where $P_{\mathrm{SUC},j}$ is the probability of successful channel reservation for the level-2 sub-network, *j*.

Substituting Equations (19)–(23) into Equation (9), the normalized throughput of the level-1 sub-network can be given as follows:

(24a) $$S_{\mathrm{net}1}=\frac{K_{\mathrm{level}-1}\delta }{5\tau_{\max}+(N_{\mathrm{level}-1}+3)\theta +N_{\mathrm{level}-1}T_{\mathrm{guard}}+\frac{K_{\mathrm{level}-1}(\delta +T_{\mathrm{guard}})}{1-p_{e}}+\bar{I}}\;$$

(24b) $$K_{\mathrm{level}-1}=\frac{N_{\mathrm{level}-1}\lambda \left[5\tau_{\max}+(N_{\mathrm{level}-1}+3)\theta +N_{\mathrm{level}-1}T_{\mathrm{guard}}+\bar{I}\right]+N_{\mathrm{trans}}\sum_{j=1}^{N_{\mathrm{net}2}}P_{\mathrm{SUC},j}N_{j}\lambda (\tau_{\max}+\theta )}{1-\frac{N_{\mathrm{level}-1}\lambda (T_{\mathrm{guard}}+\delta )}{1-p_{e}}}\;$$

(24c) $$\bar{I}=\underset{j}{\max}N_{\mathrm{trans}}\left(\left[6\tau_{\max}+(N_{j}+4)\theta +\frac{N_{j}\lambda (\tau_{\max}+\theta )(T_{\mathrm{guard}}+\delta )}{1-p_{e}}\right]P_{\mathrm{SUC},j}+(\tau_{\max}+\theta )(1-P_{\mathrm{SUC},j})\right)\;$$

(24d) $$P_{\mathrm{SUC},j}=\frac{N_{j}\lambda (\tau_{\max}+\theta )e^{-\lambda (N_{j}+N_{\mathrm{int},j})(\tau_{\max}+\theta )}}{1-e^{-N_{j}\lambda (\tau_{\max}+\theta )}}\;$$

## 6. Numerical Results and Discussions

**Figure 10:**
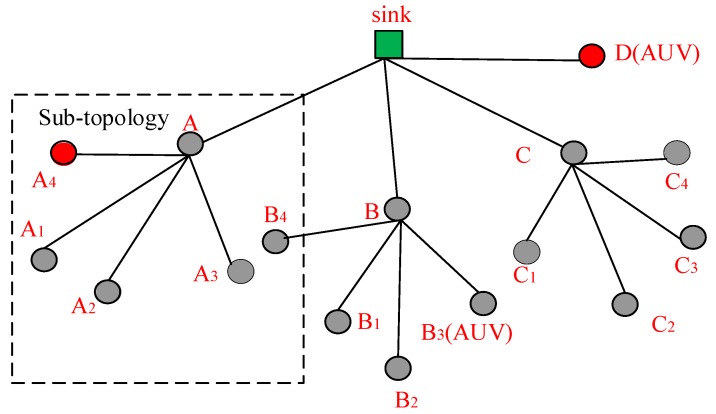
Network topology used in simulations.

We use Aqua-sim [23] based on NS-3 to evaluate the performance of the proposed DCO-MAC protocol, and compare the DCO-MAC protocol with some existing underwater MAC protocols. The network topology used in the simulations, as shown in Figure 10, is a tree-topology-based UASN, where the level-1 nodes and level-2 nodes are distributed in the transmission range of the sink node and the corresponding level-1 nodes, respectively.

The simulation parameters are listed in Table 1.

Firstly, we compare the performance of the CT-MAC protocol, in terms of the normalized throughput and the packet delay, with two contention-based protocols, slotted floor acquisition multiple access (SFAMA) and T-Lohi. Three contention-based protocols are performed in a sub-topology, as illustrated in Figure 10, which consists of a level-1 node A; four level-2 nodes, A_1_, A_2_, A_3_, A_4_; and an interference node, B_4_.

**Figure 11:**
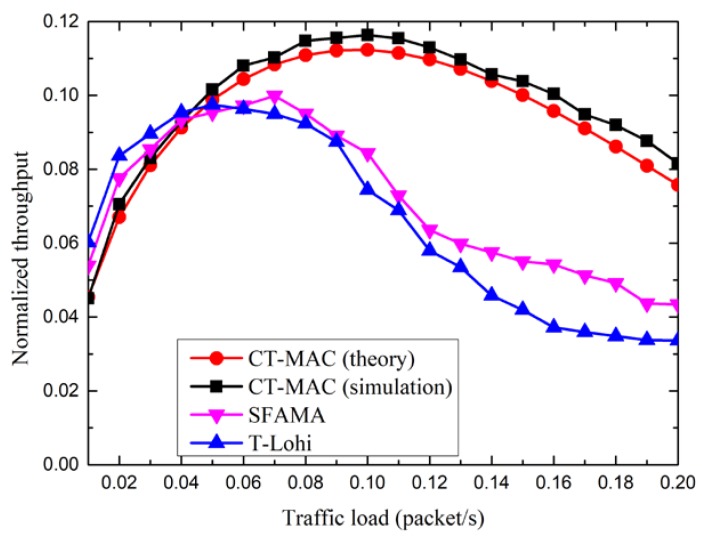
The normalized throughput of the CT-MAC protocol in sub-topology.

Figure 11 shows the normalized throughput of three MAC protocols, CT-MAC, SFAMA, and T-Lohi. From Figure 11, we observe that, for the normalized throughput of the CT-MAC protocol, the simulation results match the analytical results relatively well, which means that the performance analysis is relatively accurate. The normalized throughput, in theory, is slightly smaller than that in the simulations. The reason for this phenomenon is that the propagation delay between any two nodes is assumed to be the maximum propagation delay in the performance analysis. Moreover, we also observe that the normalized throughput of the three MAC protocols increases first as the traffic load increases. After a peak value is reached, the normalized throughput decreases along with the increase of the traffic load. The reason for this phenomenon is that these three MAC protocols are contention-based. When the traffic load is large enough, the probability of a successful channel reservation decreases as the traffic load increases.

Furthermore, the normalized throughput of the CT-MAC protocol is smaller than that of the SFAMA and T-Lohi protocols when the traffic load is light. The reason for this is that the level-1 node should spend some time to invite the other level-2 nodes in order to join in the transmission after a successful channel reservation using an RTS packet in the CT-MAC protocol. When the traffic load is light, the number of level-2 nodes with data packet(s) to be transmitted is small. A few extra data packets are added as more channel reservation time is used. Hence, the normalized throughput of the CT-MAC protocol is relatively small. However, the normalized throughput of the CT-MAC protocol is larger than that of the SFAMA and T-Lohi protocols when the traffic load is large. The reason for this phenomenon is that the level-1 node receives data packets from more than one level-2 node, with data packet(s) to be transmitted after a successful channel reservation in the CT-MAC protocol.

**Figure 12:**
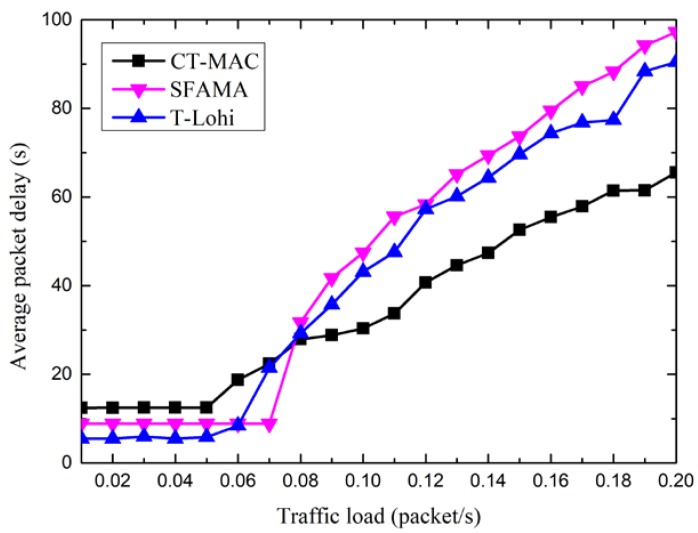
The average packet delay of the CT-MAC protocol in sub-topology.

Figure 12 shows the packet delay of three MAC protocols, CT-MAC, SFAMA, and T-Lohi. From Figure 12, we observe that the packet delay of the three MAC protocols increases as the traffic load increases. After the traffic load reaches a specific value, the packet delay increases rapidly along with the increase of the traffic load. The reason for this phenomenon is that these three MAC protocols are contention-based. When the traffic load is large enough, the probability of a successful channel reservation decreases as the traffic load increases. Without the reserved channel resource, the data packets in the level-2 nodes should wait to transmit, and the packet delay therefore increases.

Moreover, the packet delay of the CT-MAC protocol is larger than that of the SFAMA and T-Lohi protocols when the traffic load is light. The reason for this is that the level-1 node spends extra time to invite other level-2 nodes to join in the transmission after a successful channel reservation in the CT-MAC protocol. When the traffic load is light, the number of extra data packets that are added is small. Hence, the packet delay of the CT-MAC protocol is relatively large. However, the packet delay of the CT-MAC protocol is smaller than that of the SFAMA and T-Lohi protocols when the traffic load is large. The reason for this phenomenon is that, after a successful channel reservation in the CT-MAC protocol, the level-1 node receives data packets from more than one level-2 node with data packet(s) to be transmitted, and the data packets in the level-2 nodes are transmitted rather than waiting in a transmission buffer.

**Figure 13:**
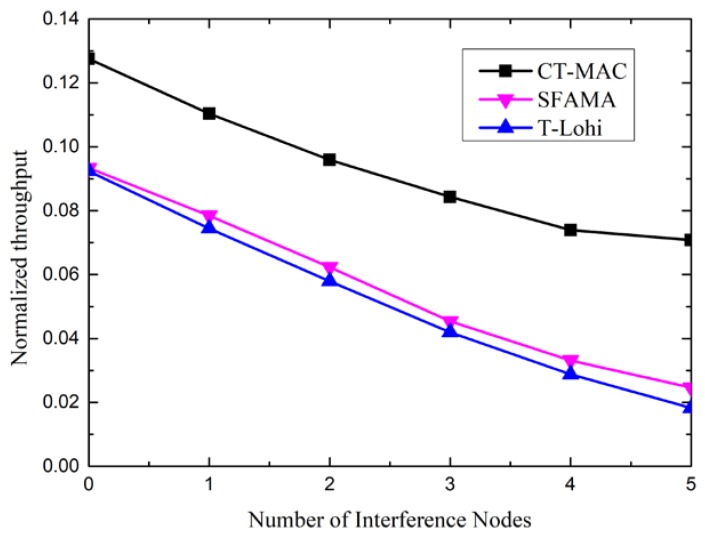
The normalized throughput of the CT-MAC protocol vs. the number of interference nodes.

To show the influence of the number of interference nodes on the performance, we changed the number of interference nodes in the sub-topology. Figure 13 shows the impact of the number of interference nodes on the performance of the CT-MAC, SFAMA, and T-Lohi protocols, in term of the normalized throughput.

From Figure 13, we observe that the normalized throughput of the three MAC protocols decreases along with the increase of the number of interference nodes. The reason is that these three MAC protocols are contention-based. The probability of successful channel reservation decreases as the number of interference nodes increases. 

Moreover, the normalized throughput of the CT-MAC protocol is larger than that of the SFAMA and T-Lohi. The reason for this is that the level-1 node receives data packets from more than one level-2 node with data packet(s) to be transmitted after a successful channel reservation in the CT-MAC protocol.

Secondly, we compare the performance of the DCO-MAC protocol, in terms of the normalized throughput, the average packet delay, energy consumption, and fairness, with three underwater MAC protocols, SFAMA, RIPT, and CH-MAC. Four MAC protocols are performed in a tree topology, as illustrated in Figure 10.

**Figure 14:**
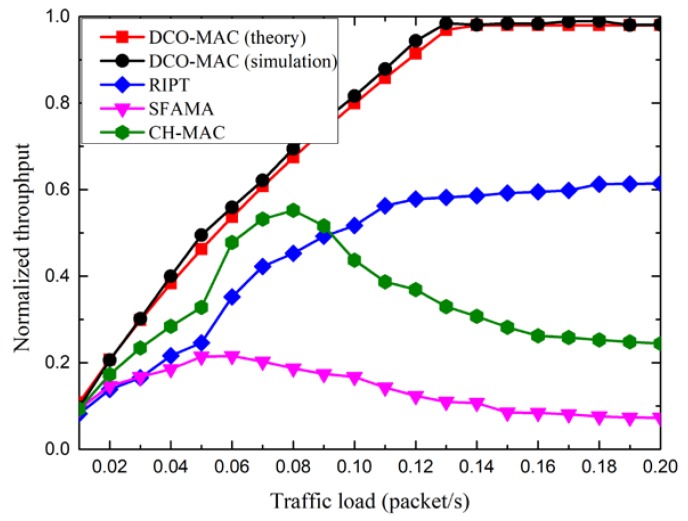
The normalized throughput of the DCO-MAC protocol.

Figure 14 shows the normalized throughput of the DCO-MAC, SFAMA, RIPT, and CH-MAC protocols. From Figure 14, we observe that, for the normalized throughput of the DCO-MAC protocol, the simulation results match the analytical results relatively well, which means that the performance analysis is accurate. The normalized throughput, in theory, is slightly smaller than that in the simulations. The reason for this phenomenon is that the propagation delay between any two nodes is assumed to be the maximum one in the performance analysis.

Moreover, as the traffic load increases, the normalized throughput of the DCO-MAC and RIPT protocols increases, while the normalized throughput of the SFAMA and CH-MAC protocols first increases and then decreases. As SFAMA is a contention-based protocol, the collision probability increases as the traffic load increases, which results in a poor performance in terms of the normalized throughput. As RIPT is a receiver-initiated protocol, the receiver schedules the data transmission of the sensor nodes to avoid the collision, which leads to a better performance. CH-MAC is a hybrid protocol, where TDMA and CSMA/CA are performed in the level-2 sub-networks and the level-1 sub-network, respectively. However, if the traffic load in the level-1 sub-network is heavier than that in the level-2 sub-network, CSMA/CA introduces a high probability of collision. Hence, the normalized throughput of the CH-MAC protocol decreases when the traffic load is large. Compared with the CH-MAC protocol, DCO-MAC uses the contention-based MAC protocol in the level-2 sub-networks with a low traffic load, so as to decrease the average packet delay, while it uses the reservation-based MAC protocol in the level-1 sub-network with a high traffic load to avoid the collision. Hence, the normalized throughput of the DCO-MAC protocol is the largest among these four MAC protocols.

**Figure 15:**
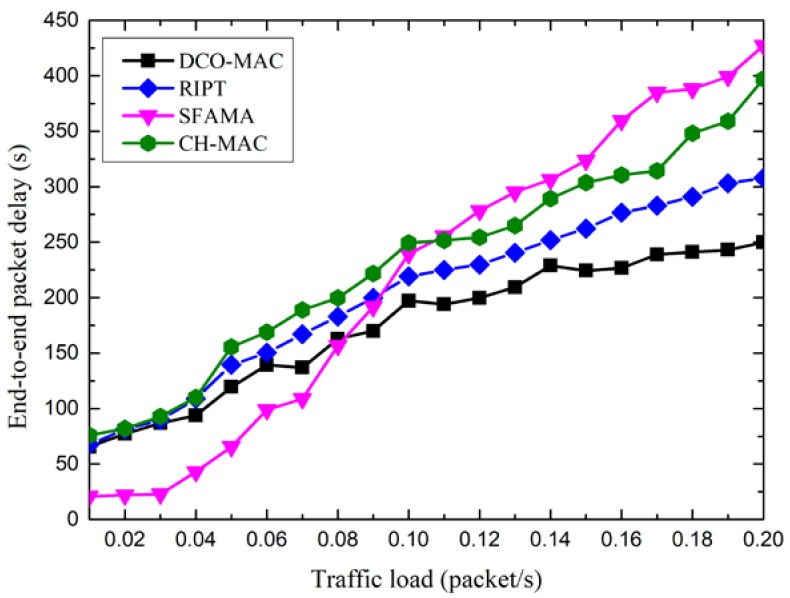
The end-to-end packet delay of the DCO-MAC protocol.

Figure 15 shows the end-to-end packet delay of the DCO-MAC, SFAMA, RIPT, and CH-MAC protocols. From Figure 15, we observe that the end-to-end packet delay of the four MAC protocols increases as the traffic load increases.

Moreover, the end-to-end packet delay of the SFAMA protocol is smallest when the traffic load is low. It is because the probability of collision is very small, and the nodes with data packets to be transmitted can always reserve a channel successfully, and the data packets are transmitted to the receiver immediately. When the traffic load increases, the probability of collision increases, and the data packets should wait a period before they can be transmitted to the receiver, which results in the increasing of the end-to-end packet delay. For the RIPT protocol, the data packets can be transmitted only when the receiver initiates the transmission, which results in a relatively large wait delay. For the CH-MAC protocol, TDMA is performed in the level-2 sub-network, and no transmission collisions happen. Although CSMA/CA is performed in the level-1 sub-network, the end-to-end delay is smaller than that of the SFAMA when the traffic load is large. For the DCO-MAC protocol, the average packet delay of the CT-MAC performed in the level-2 sub-networks is relatively small, as shown in Figure 12. In addition, a reservation-based MAC protocol is performed in the level-1 sub-network to avoid the collision. Therefore, the end-to-end packet delay is smallest among the four MAC protocols.

**Figure 16:**
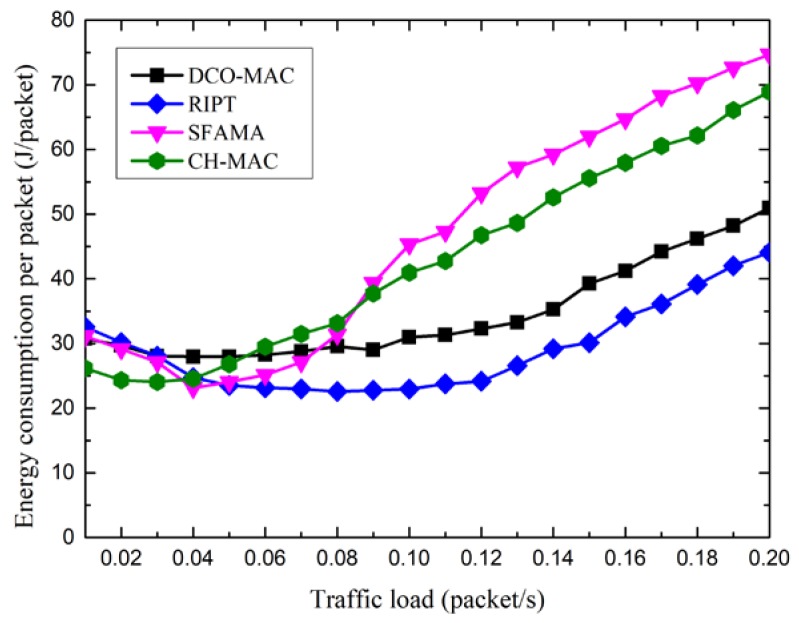
The energy overhead of the DCO-MAC protocol.

Figure 16 shows the energy overhead of the DCO-MAC, SFAMA, RIPT, and CH-MAC protocols, where the energy overhead is defined as the energy consumption per data packet transmission. From Figure 16, as the traffic load is low, the energy consumption of the four MAC protocols decreases slightly along with the increase of the traffic load. The reason for this is that the number of data packets that are successfully transmitted increases as the traffic load increases. After a minimum value is reached, the energy overhead increases along with the increase of the traffic load. The reason for this is that as the traffic load is large, and the probability of collision increases along with the traffic load, which results in the increase of energy overhead for the contention-based MAC protocol. As SFMA, CAMA/CA in CH-MAC, and CT-MAC in DCO-MAC are contention-based protocols, the energy overhead of these three MAC protocols increases along with the increase of the traffic load. For the RIPT protocol, the receiver schedules the data transmission to avoid collision at the receiver. Hence, the energy overhead is the lowest among the four MAC protocols.

From Figure 16, we also observe that the energy overhead of the DCO-MAC is lower than that of the SFAMA and CH-MAC protocols. The reason for this phenomenon is that, in the DCO-MAC protocol, the contention-based MAC protocol is performed in the level-2 sub-networks with a low traffic load, and a reservation-based protocol is used to avoid the collision in the level-1 sub-network with a high traffic load. Hence, the collision probability in the DCO-MAC is smaller than that of SFAMA and CH-MAC, which results in a lower energy overhead.

Fairness affects the network survival time, and is an important performance metrics of the MAC protocol. Here, using Jain’s fairness index (FI) [20], the fairness of MAC protocols is defined as follows:

(25) $$\mathrm{FI}=\frac{{\left(\sum_{i=1}^{n}m_{i}\right)}^{2}}{n\sum_{i=1}^{n}m_{i}^{2}}\;$$

where *n* denotes the number of nodes, and *m_i_* denotes the number of data packets transmitted by node *i* during the simulation period. The value of FI ranges from 0 to 1. FI = 1 if the number of data packets transmitted by all of the nodes is the same, which means that the fairness of the considered MAC protocol is the best.

**Figure 17:**
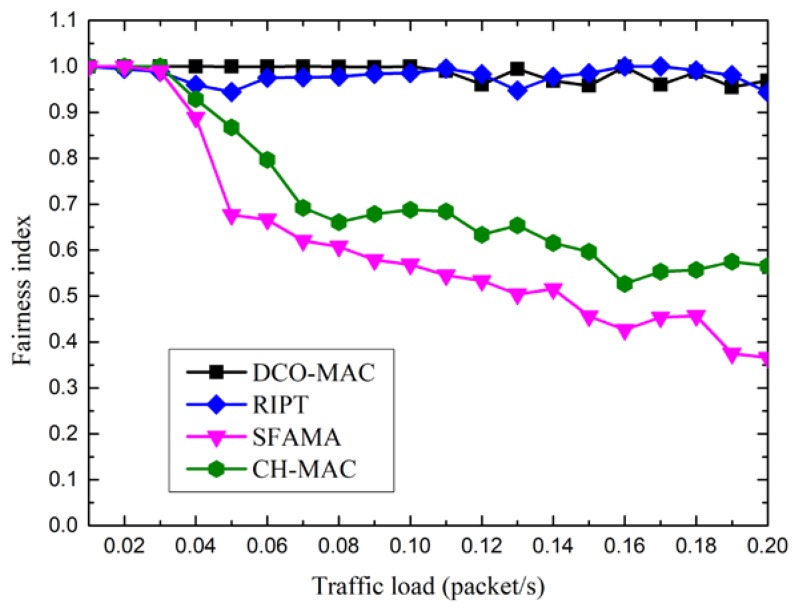
The fairness index of the DCO-MAC protocol.

Figure 17 shows the fairness index of the DCO-MAC, SFAMA, RIPT, and CH-MAC protocols. From Figure 17, we observe that the fairness index of the SFAMA and CH-MAC protocols decreases as the traffic load increases. In conventional MAC protocols, the distance between the two nodes is a key factor in the channel competition because of the large propagation in the UASN. That is, the sender located near the receiver has a high channel access probability. Hence, the fairness of the contention-based MAC protocols, such as SFAMA and CSMA/CA in CH-MAC, is low. Although CT-MAC in DCO-MAC is contention-based, the level-1 node can invite other level-2 nodes to join in the transmission after a successful channel reservation, which means that the level-2 nodes have the same opportunity to transmit data packets, regardless of their locations. Moreover, as RSV-MAC in DCO-MAC is receiver-initiated, the receiver schedules the senders’ transmission in a fair manner. Therefore, the DCO-MAC protocol has a good fairness performance. As a receiver-initiated protocol, the fairness of the RIPT protocol also performs well.

**Figure 18:**
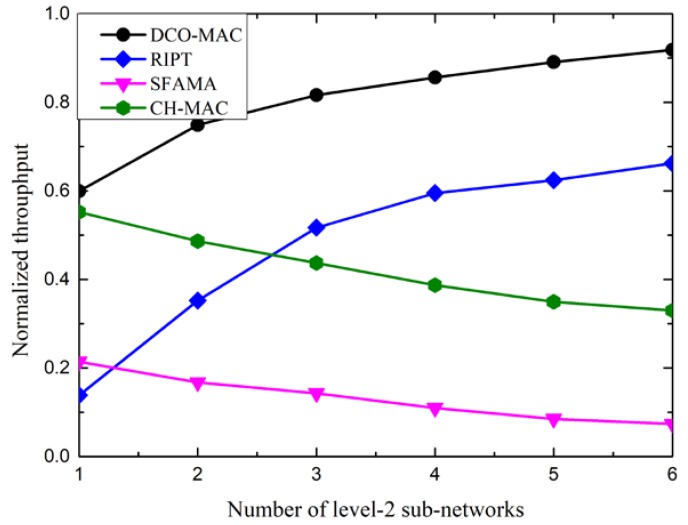
The normalized throughput of the DCO-MAC protocol vs. the number of level-2 sub-networks.

To show the influence of the number of level-2 sub-networks on the performance, we changed the number of level-2 sub-networks in the simulation topology. Figure 18 shows the impact of the number of level-2 sub-networks on the performance of the DCO-MAC, SFAMA, RIPT, and CH-MAC protocols, in terms of the normalized throughput.

From Figure 18, we observe that the normalized throughput of the SFAMA and CH-MAC protocols decreases as the number of level-2 sub-networks increases. As SFAMA and CSMA/CA in CH-MAC are contention-based protocols, the collision probability in the level-1 sub-network increases along with the increase of the number of level-2 sub-networks, which results in the performance degradation. As RIPT and RSV-MAC in DCO-MAC are receiver-initiated protocols, the receiver schedules the data transmission of the sensor nodes to avoid the collision. The traffic load in the level-1 sub-networks increases along with the increase of the number of level-2 sub-networks. Therefore, the normalized throughput of the receiver-initiated protocols increases.

## 7. Conclusions

In this paper, we proposed a hybrid MAC protocol in a UASN for data collection. We focused on the data collection applications that make the traffic load non-uniformly distributed in the network. According to the traffic load, the network is partitioned into two kinds of sub-networks, and different MAC protocols are carried out in the different sub-networks. A modified contention-based MAC protocol is used in the sub-network with a light traffic load, to improve the performance, and a reservation-based MAC protocol is used in the sub-network with heavy traffic load, the to avoid the transmission collision. In addition, the proposed MAC protocol considered the access of mobile nodes performing the data collection task. Theoretical analysis and simulation results demonstrate that the modified contention-based MAC (CT-MAC) protocol outperforms the conventional contention-based MAC protocols for data collection in the UASN, in terms of the normalized throughput and the average packet delay. Moreover, the proposed MAC protocol performs well in the DCO-UASN, with, for example, a large normalized throughput, a low end-to-end packet delay, a small energy overhead, and a high fairness.

In the future research, we will extend this work to a larger UASN with a more complicated traffic load distribution. On the other hand, we will validate the proposed DCO-MAC protocol in the real-life underwater acoustic communication transmission scenarios.

## Figures and Tables

**Table 1:** Simulation parameters.

Parameters	Value
Control packet length	20 Bytes
Data packet length	200 Bytes
Bit error rate	10^−5^
Guard time	0.02 s
Maximum transmission range	3 km
Transmission rate	800 bps
*N*_trans_	2
Speed of mobile nodes	2.5 m/s
Transmit power	10 W
Receive power	0.4 W
Listen power	0.08 W
Idle power	0.08 W

## References

[1] Akyildiz I.F., Pompili D., Melodia T. (2005). Underwater acoustic sensor networks: Research challenges. Ad Hoc Netw..

[2] Chitre M., Shahabudeen S., Stojanovic M. (2008). Underwater acoustic communications and networking: Recent advances and future challenges. Mar. Technol. Soc. J..

[3] Heidemann J., Stojanovic M., Zorzi M. (2012). Underwater sensor networks: Application, advances and challenges. Philos. Trans. R. Soc. A.

[4] Vieira L.F.M., Kong J., Lee U. Analysis of ALOHA protocols for underwater acoustic sensor networks. Proceedings of the ACM WUWNet’06.

[5] Ng H., Soh W.S., Motani M. MACA-U: A media access protocol for underwater acoustic networks. Proceedings of the IEEE GLOBECOM 2008.

[6] Molins M., Stojanovic M. Slotted FAMA: A MAC protocol for underwater acoustic network. Proceedings of the IEEE/MTS OCEANS 2006.

[7] Syed A.A., Ye W., Heidemann J. (2008). Comparison and evaluation of the T-Lohi MAC for underwater acoustic sensor networks. IEEE J. Sel. Areas Commun..

[8] Ng H., Soh W.S., Motani M. BIC-MAC: Bidirectional-concurrent MAC protocol with packet bursting for underwater acoustic networks. Proceedings of the IEEE/MTS OCEANS 2010.

[9] Guo X., Frater M.R., Ryan M.J. (2009). Design of a propagation-delay-tolerant MAC protocol for underwater acoustic sensor networks. IEEE J. Ocean. Eng..

[10] Lin W., Chen K. (2016). MHM: A multiple handshaking MAC protocol for underwater acoustic sensor networks. Int. J. Distrib. Sens. Netw..

[11] Chen H., Fan G., Xie L. (2013). A hybrid path-oriented code assignment CDMA-based MAC protocol for underwater acoustic sensor networks. Sensors.

[12] Liao Z., Li D. (2015). A handshake based ordered scheduling MAC protocol for underwater local area networks. Int. J. Distrib. Sens. Netw..

[13] Chirdchoo N., Soh W.S., Chua K.C. (2008). RIPT: A receiver-initiated reservation-based protocol for underwater acoustic networks. IEEE J. Sel. Areas Commun..

[14] Shahabudeen S., Chitre M. (2014). Adaptive multimode medium access control for underwater acoustic networks. IEEE J. Ocean. Eng..

[15] Zhu M., Zhang W., Jin N. (2016). UPMAC: A localized load-adaptive MAC protocol for underwater acoustic networks. IEEE Sens. J..

[16] Lotfinezhad M., Liang B., Sousa E.S. (2008). Adaptive cluster-based data collection in sensor networks with direct sink access. IEEE Trans. Mob. Comput..

[17] Yoon S., Qiao C. A new search algorithm using autonomous and cooperative multiple sensor nodes. Proceedings of the IEEE INFOCOM 2007.

[18] Khan J.U., Cho H.S. (2016). Data-gathering scheme using AUVs in large-scale underwater sensor networks: A multi-hop approach. Sensors.

[19] Jagannath J., Saji A., Kulhandjian H. A hybrid MAC protocol with channel-dependent optimized scheduling for clustered underwater acoustic sensor networks. Proceedings of the ACM WUWNet’13.

[20] Hollinger G.A., Choudhary S., Qarabaqi P. Communication protocols for underwater data collection using a robotic sensor network. Proceedings of the IEEE GLOBECOM 2011.

[21] Deng M., Chen H., Xie L. A hybrid MAC protocol in data-collection-oriented underwater acoustic sensor networks. Proceedings of the IEEE/MTS OCEANS 2017.

[22] Fullmer C.L., Garcia-Luna-Aceves J. Floor acquisition multiple access for packet-radio networks. Proceedings of the ACM SIGCOMM 1995.

[23] Zhu Y., Lu X., Pu L. Aqua-sim: An NS-2 based simulator for underwater sensor networks. Proceedings of the ACM WUWNet’13.

